# The Neighborhood Effect Averaging Problem (NEAP): An Elusive Confounder of the Neighborhood Effect

**DOI:** 10.3390/ijerph15091841

**Published:** 2018-08-27

**Authors:** Mei-Po Kwan

**Affiliations:** Department of Geography and Geographic Information Science, Natural History Building, 1301 W Green Street, University of Illinois at Urbana-Champaign, Urbana, IL 61801, USA; mpk654@gmail.com

**Keywords:** the neighborhood effect averaging problem (NEAP), human mobility, environmental exposure, the uncertain geographic context problem, UGCoP

## Abstract

Ignoring people’s daily mobility and exposures to nonresidential contexts may lead to erroneous results in epidemiological studies of people’s exposures to and the health impact of environmental factors. This paper identifies and describes a phenomenon called neighborhood effect averaging, which may significantly confound the neighborhood effect as a result of such neglect when examining the health impact of mobility-dependent exposures (e.g., air pollution). Several recent studies that provide strong evidence for the neighborhood effect averaging problem (NEAP) are discussed. The paper concludes that, due to the observed attenuation of the neighborhood effect associated with people’s daily mobility, increasing the mobility of those who live in disadvantaged neighborhoods may be helpful for improving their health outcomes.

The neighborhood effect is a central analytic notion in epidemiological studies for assessing people’s exposures to and the health impact of environmental factors. Past studies have largely used the residential neighborhood, often operationalized as static administrative areas such as the home census tract, as the contextual area to examine people’s environmental exposures. For health behaviors or outcomes that are heavily influenced by environmental factors in a person’s residential neighborhood or the areas nearby (e.g., social capital and collective efficacy at the neighborhood level), this approach may be adequate. However, using this residence-based approach may lead to erroneous results for health outcomes that are also influenced by exposures to environmental factors in neighborhoods other than the residential neighborhood (e.g., air pollution) because most people move around to undertake their daily activities and come under the influence of many different neighborhood contexts outside their home neighborhoods [1,2,3,4].

As recent studies have shown, ignoring people’s daily mobility and exposures to nonresidential contexts may lead to erroneous results. For instance, Park and Kwan [5] compared individual air pollution exposures in Los Angeles (CA, USA) using four combinations of spatial and temporal attributes: residence-based hourly levels, residence-based daily levels, mobility-based hourly levels, and mobility-based daily levels (where an hourly exposure level was estimated using cokriging for each of the 24 h of a day while the daily level was the average of these 24 hourly exposure levels for the day). The results indicated that these four exposure estimates are significantly different, suggesting that individual exposures may be under- or over-estimated if human mobility and the spatiotemporal variability of air pollution levels are not taken into account. The study argued that ignoring human mobility may lead to misleading results in air pollution studies. Another study in Israel by Shafran-Nathan et al. [6] observed that differences between home-based and work-based exposures to nitrogen oxides are considerable for over 50% of the subjects, and it is equally likely that a subject’s residence-based exposure is either higher or lower than the mobility-based exposure (which takes into account a subject’s work/school location). The study concluded that estimating air pollution exposures at subjects’ home location may under- or overestimate exposures when compared to exposure estimates that take their daily mobility into account.

Several recent studies not only provide evidence on how ignoring daily human mobility may lead to misleading results in exposure and health impact assessments but also highlight a specific reason that contributes to estimation errors [6,7,8]. This phenomenon may be called neighborhood effect averaging, which operates as follows.

Given that most people move around to undertake their daily activities (e.g., shop, attend school, or go to work), they are exposed to the environmental contexts of many different areas outside their residential neighborhoods in the course of a day. As a person travels to areas outside of his or her residential neighborhood, the person may experience similar or different levels of exposure when compared to that of his or her residential neighborhood. Because of the diversity in the intensity of the environmental factor in question (e.g., air pollution) over space in any study area, a person’s exposure level in nonresidential neighborhoods may be higher, lower, or similar when compared to the exposure level experienced in his or her residential neighborhood. As indicated by recent studies, the probability distribution of individual residence-based exposure approximates a bell-shaped distribution, which means that many people have exposure levels around the mean value for the population of the study area, while fewer people have very high or low exposure levels [6,7,8]. Therefore, a person who lives in a neighborhood with a high level of an environmental factor (and thus exposure) will visit areas that are more likely to have lower levels of such environmental factor as a result of his or her daily mobility, while a person who lives in a neighborhood with a low level of the environmental factor will visit areas that are more likely to have higher levels of such environmental factor. For those who have residence-based exposure levels around the mean value, their exposure levels in nonresidential neighborhoods tend to be similar to those of their residential neighborhoods because they will visit areas that are less likely to have significantly different levels of such environmental factor in their daily life.

As a result, the neighborhood effect assessed with a traditional residence-based approach for individuals whose residence-based exposures are much higher than the mean exposure will be overestimated because these individuals tend to experience lower levels of exposure outside their residential neighborhoods, which attenuates their high exposures in their residential neighborhoods [7,8]. On the other hand, the neighborhood effect for individuals whose residence-based exposures are much lower than the mean exposure will be underestimated because these individuals tend to experience higher levels of exposure outside their residential neighborhoods, which moderates their low exposures in their residential neighborhoods [7,8].

Taking people’s daily mobility into account (which will generate more accurate assessments for mobility-dependent exposures) will therefore lead to an overall tendency toward the mean exposure because exposure levels for people whose residence-based exposures are lower or higher than the mean exposure will tend toward the mean exposure, thus moderating the influence of the environmental factor in their residential neighborhoods on their health behaviors or outcomes. This is neighborhood effect averaging. It means that for health outcomes that are also affected by exposures to environmental factors in people’s nonresidential neighborhoods as they move around in their daily life (mobility-dependent exposures), using residence-based neighborhoods to estimate individual exposures to and the health impact of environmental factors will tend to overestimate the statistical significance and effect size of the neighborhood effect because it ignores the confounding effect of neighborhood effect averaging that arises from human daily mobility. This is a fundamental methodological issue when examining the health effects of mobility-dependent exposures and may be called the neighborhood effect averaging problem (NEAP).

Two recent studies have observed the phenomenon of neighborhood effect averaging. A study in Belgium found that exposures to NO_2_ for mobile phone users with low residence-based NO_2_ exposures are 54.5% higher when their daily mobility is taken into account, while exposures to NO_2_ for mobile phone users with high residence-based NO_2_ exposures are 33.1% lower when their daily mobility is taken into account [7]. Another study in China found that residence-based estimates of individual exposures to six air pollutants (carbon monoxide, nitrogen dioxide, sulfur dioxide, ozone, particulate matter with aerodynamic diameter less than 2.5 µm [PM_2.5_], and elemental carbon) tend to overestimate exposures for people with high residence-based exposures and underestimate exposures for people with low residence-based exposures [8]. The study also observed that the range between the maximum and minimum as well as the 5th and 95th percentile exposure estimates is smaller for the mobility-based approach than for the residence-based approach, indicating that individual exposures are less variable when people’s mobility is taken into account. These two studies thus provide strong evidence for the neighborhood effect averaging problem (NEAP) when assessing individual-based mobility-dependent exposures.

However, given that these studies are both on air pollution and cover only two study areas, further evidence is needed for assessing whether the NEAP also holds true for mobility-dependent exposures other than air pollution and in other study areas. It is also important to note that while the probability distribution of individual residence-based exposure approximates a bell-shaped distribution [7,8], it may be a normal or non-normal (i.e., skewed) distribution. For instance, the distribution of individual residence-based exposures across a city may be skewed and non-normal as a result of the particular geographic distribution of its population and specific environmental factors. However, even when the probability distribution of individual residence-based exposures is a non-normal bell-shaped distribution, many people would still have exposure levels close to the mean value for the population, and fewer people would have very high or low exposure levels as far as such distribution is slightly to moderately skewed. As a result, it is likely that neighborhood effect averaging would hold for slightly to moderately skewed distributions of individual residence-based exposures across a study area; but again, further evidence is needed to evaluate the extent to which this is true.

Further, neighborhood effect averaging may operate over different time scales: exposures to different neighborhood contexts via a person’s daily mobility may reduce the influence of the residential context, while the effect of exposure to a person’s current residential neighborhood may be mitigated by exposures to the residential contexts experienced earlier in life (e.g., childhood or previous residences). Considering the effects of daily human mobility and mobility over the life course (residential mobility and migration) is thus essential in certain epidemiological studies, especially when examining environmental contexts that are highly mobility-dependent (e.g., air pollution, noise pollution, healthy food outlets, green spaces, and cancer risk) [3,4,9].

The implications of neighborhood effect averaging for public health policies is that increasing the mobility of those who live in disadvantaged neighborhoods through better, safer, and more reliable public transit, in addition to improving neighborhood quality *in situ*, may be helpful for improving their health outcomes. Recent studies on the activity spaces and segregation experiences of disadvantaged social groups found that people who live in highly segregated neighborhoods tend to work or conduct many of their daily activities in relatively integrated urban areas (when compared to their residential neighborhoods) [10,11]. This observation supports the view that increasing the daily mobility of marginalized social groups or racial minorities to diverse parts of an urban area may mitigate the social disadvantages they experience, including disproportionate exposures to health risks [10,11]. However, as Wang et al. [12] recently found, even when residents of disadvantaged neighborhoods regularly travel to advantaged neighborhoods, their relative isolation and segregation may persist. Thus, concerted policies of social integration that mitigate racial discrimination and reduce segregation are ultimately critical, and more research on the positive and negative implications of neighborhood effect averaging is sorely needed.
